# Enhancing Badminton Game Analysis: An Approach to Shot Refinement via a Fusion of Shuttlecock Tracking and Hit Detection from Monocular Camera

**DOI:** 10.3390/s24134372

**Published:** 2024-07-05

**Authors:** Yi-Hua Hsu, Chih-Chang Yu, Hsu-Yung Cheng

**Affiliations:** 1 Department of Information and Computer Engineering, Chung Yuan Christian University, Taoyuan City 320314, Taiwan; 2 Department of Computer Science and Information Engineering, National Central University, Taoyuan City 320317, Taiwan; chengsy@csie.ncu.edu.tw

**Keywords:** small object tracking, action detection, sports analytics

## Abstract

Extracting the flight trajectory of the shuttlecock in a single turn in badminton games is important for automated sports analytics. This study proposes a novel method to extract shots in badminton games from a monocular camera. First, TrackNet, a deep neural network designed for tracking small objects, is used to extract the flight trajectory of the shuttlecock. Second, the YOLOv7 model is used to identify whether the player is swinging. As both TrackNet and YOLOv7 may have detection misses and false detections, this study proposes a shot refinement algorithm to obtain the correct hitting moment. By doing so, we can extract shots in rallies and classify the type of shots. Our proposed method achieves an accuracy of 89.7%, a recall rate of 91.3%, and an F1 rate of 90.5% in 69 matches, with 1582 rallies of the Badminton World Federation (BWF) match videos. This is a significant improvement compared to the use of TrackNet alone, which yields 58.8% accuracy, 93.6% recall, and 72.3% F1 score. Furthermore, the accuracy of shot type classification at three different thresholds is 72.1%, 65.4%, and 54.1%. These results are superior to those of TrackNet, demonstrating that our method effectively recognizes different shot types. The experimental results demonstrate the feasibility and validity of the proposed method.

## 1. Introduction

Recently, the importance of scientific training methods and data analysis has notably increased in professional sports. Typically, the analyzed information includes important knowledge related to sports training; both coaches and athletes develop improved training methods through the acquired information, thereby achieving superior results in competitions. However, its effectiveness can be hard to observe due to information gathering being a multifaceted domain. It requires a deep understanding of advanced training theories and methodologies from sports-dominant nations, the incorporation of innovative skills and tactics, cutting-edge training equipment, and the comprehension of opponent characteristics and their habits. If these requirements can be met, the provided information allows athletes to better realize the impact of their training intensity and effectiveness. Furthermore, it helps them understand and apply tactical skills that specifically match their opponents’ traits [1].

To illustrate how data analysis impacts the sports domain, this study initially discusses its applications across various sports fields. Data analysis has been successfully applied in Major League Baseball (MLB) for many years—for example, the Oakland Athletics, a team with a limited budget and no superstars, whose manager evaluated players based on their on-base percentage (OBP) rather than traditional statistics like batting average (BA) or on-base plus slugging (OPS). This strategy led them to win many games and become a legendary story in baseball. Apart from that, the results of data analysis also provide clear and strong evidence to break the myth of the “Curse of the Bambino” or the “Curse of the Billy Goat” [2,3,4].

Nowadays, many existing systems, such as Trackman [5,6] and Rapsodo [7,8], are utilized for obtaining imperceptible data in the game. Huang and Hsu [9] analyzed 5297 pitching videos from the 2018 Chinese Professional Baseball League using Trackman, examining each umpire’s strike zone under different conditions, such as different ball counts and batters. On the other hand, Rapsodo tracks the trajectory of a pitch through a single camera and flashes infrared to calculate the spin rate, horizontal and vertical break, etc. It can also be used for tracking a batter’s swing, providing statistics such as the exit velocity, launch angle, and exit direction. The main difference between Trackman and Rapsodo is the provided data format; the former provides 2D data, while the latter provides 3D data. The application of these systems provides teams with more comprehensive and advanced information, which helps players and coaches understand and devise game strategies.

In the field of basketball, where top teams often have similar physical capabilities and motivation levels, data analysis outcomes could be an important reference for team victories and determining training approaches [10,11]. One way to collect data from a basketball court is by using the SportVU multi-camera tracking system. This system tracks player positions and records every dribble, pass, shot, and touch, thereby providing rich statistics, including running distances, attempted shots, fouls committed, shooting efficiency, etc. [12,13]. Over the past few years, the NBA has been using SportVU to provide statistics to its audience via its website and streaming platforms. Meanwhile, teams also utilize SportVU to evaluate player abilities, which is an important factor for estimating their contributions [14,15]. After acquiring these statistics, many studies applied deep learning techniques for different purposes, such as analyzing factors that affect shooting accuracy, classifying offensive tactics, and predicting game results [16,17,18]. In order to retain players’ physical fitness, team owners invested heavily in sports medicine to improve their training efficiency. However, millions in actual and potential income were still lost due to athlete injuries. To address this problem, Cohan et al. collected historical data on player injuries from a public dataset and developed the deep learning model, METIC, thereby allowing players and managers to predict potential injuries according to players’ past injuries, game activities, and statistics, thereby reducing the chances of player injuries [19].

SportVU, along with ProZone [20], OPTA [21], and DeltaTre [22], is used in the field of soccer as a major data supplier. These systems utilize multiple infrared and high-speed cameras to track players and the ball. This allows for the evaluation of advanced metrics like ground duels, dribble pasts, expected assists, pass completion rates, and the number of key passes. These statistics go beyond traditional measurements such as goals and saves, offering novel insights into players’ true abilities. Coaches can use this information to identify those players who may not score much but contribute significantly to the game with accurate passes and tackles. This helps in determining players suitable for core offensive roles and those who excel defensively, resulting in more effective lineup arrangements and tactical strategies [23,24,25]. In addition, researchers also use these data with deep learning for event detection, for example, determining whether the player is passing or shooting the ball. This approach could be employed to predict the defender’s next move during offense or even the game’s outcome [26,27,28].

In badminton, details such as player movement and swing speeds, often unnoticed by the naked eye, are typically extracted from high-speed cameras and motion-tracking systems. This information gives players a deeper understanding of their skill level and helps coaches and technical teams analyze the strengths and weaknesses of players, thus enabling them to devise more effective training plans. Further, the analysis can be applied to opponents to identify their strengths and weaknesses, leading to the development of corresponding counter-strategies [29,30,31].

In these studies, we can observe the significant impact of data science on various sports disciplines. The success of data analysis depends on big data, but traditional methods of collecting valuable information are labor-intensive and require an extensive review of extensive game data. Without this process, it is challenging for experts to summarize valuable insights into players’ characteristics, strengths, and weaknesses. Even when information is collected, organizing these data to create effective training plans is time-consuming. Therefore, the need for accuracy, efficiency, and scalability drives the development of an automated information-gathering system, which is also the aim of this study.

To achieve the above goal, this study presents an automated analytical system that analyzes badminton games from a monocular camera. This system focuses on accurately identifying the rallies and extracting each shot between two players by tracking the shuttlecock and players’ movements on the court. Features such as types of shots, the average movement distance, and scoring area distribution can help players understand opponents’ tactics. They can utilize this information as a reference to improve their on-court performance and increase their chances of winning.

The objective of this study is to provide a data-driven understanding of individual player abilities for both players and coaches, thereby propelling the development of training plans and competitive strategies. Leveraging computer vision and machine learning technologies, we aim to tackle the challenge of acquiring more accurate shots from regular competition videos. This approach allows us to conduct an in-depth analysis of individual playing styles, helping the coach to understand each player’s unique characteristics and enabling the customization of specific training plans. While this methodology is applied to badminton, it also holds the potential for transferability to a wide range of other sports disciplines.

The contributions of this study are as follows:

1. We propose a shot refinement algorithm that uses the results of shuttlecock tracking and action detection, demonstrating improved results compared to previous methods.
2. The extracted shots are more precise than those obtained using previous methods, particularly in circumstances with numerous detection misses of the shuttlecock. This leads to improved accuracy in shot-type classification.
3. The proposed method only analyzes videos captured from a monocular camera, making it applicable to existing badminton videos without any additional hardware.

The rest of this paper is organized as follows. Section 2 provides related works in sports analytics, particularly focusing on computer vision approaches. Section 3 describes the proposed algorithm and modules used in this study. Section 4 highlights some experiments performed to prove the feasibility and superiority of the proposed method, followed by conclusions in Section 5.

## 2. Related Work

### 2.1. Sports Analytics with Computer Vision and Deep Learning

Numerous studies have leveraged computer vision technology to facilitate automated sports analysis. For example, a real-time basketball shooting spot analysis system was designed using deep neural networks (DNNs) [32]. The system analyzes basketball videos from a court view, tackling challenges such as the panning and zooming of the camera, and is capable of accurately identifying the exact timepoint of a shot. The study predicted the types of shots to be a three-pointer, two-pointer, or free throw. Some studies utilize a Virtual Reality (VR) system to provide an immersive environment. This has been developed to enhance court vision for basketball athletes, which is a critical competency in competitive scenarios. Enhanced court vision enables athletes to effectively identify teammates with open shot opportunities, observe the positioning of defensive players around the open space, and promptly select appropriate passing paths. A practical example of this approach is VisCoach [33], which simulates the player’s viewing perspective and provides high-quality visual training tasks to expedite the player’s development process. By aligning the court situation with the player’s eye gaze, the system can analyze vision-related behaviors to evaluate the effectiveness of the training.

DNNs have also found significant applications in the field of football, particularly in predicting match outcomes [27]. The employed dataset encompasses a comprehensive range of factors, including team rankings, past performances, and the results of previous international football matches, among other relevant variables. By deploying a DNN to explore and process this vast array of sports data, predictive values can be generated. As per the proposed DNN architecture, the model demonstrated exceptional performance in predicting the outcomes of the FIFA 2018 World Cup matches, achieving a remarkable accuracy rate of 63.3%.

Wang et al. proposed an innovative system, Coach AI [34], which utilizes deep learning methodologies for the analysis of badminton game videos. This system effectively extracts vital information such as player’s movements, positioning, and strategies. Moreover, it is capable of detecting the trajectory of the shuttlecock, thereby enabling the identification of the players’ swing moments, segmentation of each round, summarization of points won and lost, and categorization of the shots. The utilization of this system significantly aids coaches and athletes in formulating strategic policies for training practice, thereby enhancing the competitive performance of the players.

### 2.2. Human Pose Estimation

In applications of motion analysis via visual-based systems, the estimation of human posture is a technique frequently employed. Plenty of techniques with high accuracy have been developed to address this complex task. Each technique presents its own unique methodologies and technical elements to effectively manage the challenges inherent in human pose estimation.

**Figure 1:**
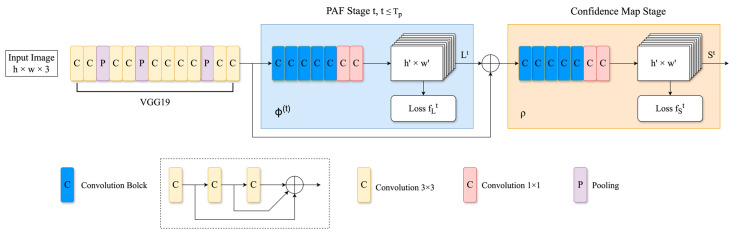
The model structure of Openpose.

A popular solution for human pose estimation is the Openpose framework [35]. Openpose is a well-known open-source library using convolutional neural networks (CNN) to achieve powerful human pose estimation. Notably, the Openpose framework is not only outstanding in estimating human poses, but it also excels at tracking facial expressions, torso, limbs, and subtle finger movements in both single- and multi-person scenarios. The network structure of Openpose is illustrated in Figure 1.

Openpose takes an image as input, followed by CNNs, forming a comprehensive feature map. These feature maps are then divided into two branches to obtain Confidence Maps and Part Affinity Fields. The use of bipartite matching helps to associate different body parts by connecting corresponding joint nodes of the individual, leveraging the nature of Part Affinity Fields for high-precision matching. This process culminates in the merging of matches, yielding a holistic skeletal representation of a person. Additionally, Openpose is able to tackle the multi-person estimation problem, treating this challenge as a graph matching problem, and navigates it with the utilization of the Hungarian Algorithm, demonstrating excellent accuracy in sophisticated multi-person pose-estimation scenarios. 

**Figure 2:**
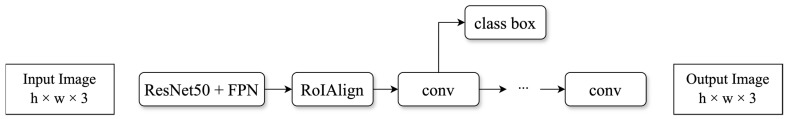
The model structure of Densepose.

Another noteworthy approach is DensePose-RCNN [36], integrated within the Detectron2 framework [37]. The DensePose-RCNN leverages three prominent deep learning models: Denspose, Mask-RCNN [38], and DenseReg [39]. The model is highly proficient in predicting dense correspondences of body parts and executing intricate pose estimations of individuals. Specifically, the model is capable of assigning each pixel to a specific part of the human body, such as the head, hands, feet, etc., and providing comprehensive posture information for these parts, including factors such as the rotation angle and position. This detailed, dense correspondence underscores the substantial application value of DensePose-RCNN. Beyond providing fundamental pose information, it aids in understanding the nuanced movements of various parts of the human body, thereby offering a wealth of potential applications in human behavior analysis and virtual try-on solutions, among others. Figure 2 depicts the network structure.

### 2.3. Multiple Object Tracking

Multi-object tracking techniques are typically used to address video-understanding problems. Currently, there are two types of solutions. The first type is the model-free method, which initially determines the positions of the objects to be tracked in the frame (usually the first frame) and then tracks these objects in subsequent frames. However, this method cannot track new or reappeared targets. Another method is tracking-by-detection. This method performs detection on every frame and then compares the detection results between them to extract the correct trajectory of each object [40,41,42,43,44,45]. For example, Bewley et al. proposed a simple online and real-time tracking (SORT) framework [41]. This method first employs Faster R-CNN with VGG16 to detect objects in the video and sets all detected targets in the first frame as tracks. Then, the Kalman filter is employed to predict the possible bounding box of each track, and matches them with the bounding box predicted by the detection model. Finally, SORT divides the matching results into three types: unmatched tracks, unmatched detections, and successful matches. A successful match means the detected bounding box and the predicted bounding box match each other, indicating that the target tracking of two consecutive frames is successful. Meanwhile, unmatched tracks are deleted, and unmatched detections are regarded as new tracks.

Due to recent advancements in deep learning techniques, tracking-by-detection methods are no longer time-consuming; thus, object-detection models are widely adopted. Apart from the Faster R-CNN used in the SORT method, another popular model is You Only Look Once (YOLO). Since time and accuracy are often contradictory, the development direction of YOLO is to make trade-offs toward speed while providing adequate detection accuracy [46,47,48,49,50,51]. In 2023, YOLOv7 [52] made significant progress in processing speed. With the same average precision as YOLOv5, the computational speed increased by 120%, making it more suitable for applications that require real-time computing. For example, Tan et al. proposed a YOLO-based multiple object tracking algorithm [53]. This algorithm uses YOLO to obtain a target’s size and position, then extracts deep features through the VGG model and inputs them into the LSTM model, thus understanding the temporal relationship between frames. Finally, objects in adjacent frames are matched by calculating the Euclidean distance between different targets. YOLO has remarkable accuracy for general objects such as the human body, but for small objects like badminton shuttlecocks, its detection accuracy is unsatisfactory. To address this problem, Cao et al. [54] proposed two novel networks based on Tiny YOLOv2, named M-YOLOv2 and YOLOBR, to enhance the shuttlecock recognition performance.

### 2.4. High-Speed Ball Tracking

For sports such as tennis, badminton, and baseball, the small size and high speed of the ball often result in blurred images in video frames, thereby posing significant challenges in tracking its trajectory [55,56,57]. To address this problem, Huang et al. proposed a deep neural network called TrackNet [58], which is specifically designed for tracking small objects moving at high speed.

**Figure 3:**
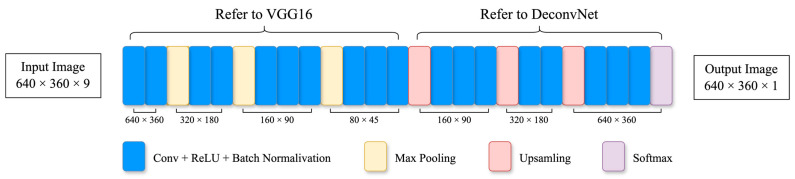
Architecture of TrackNet.

As shown in Figure 3, TrackNet adopts the Convolutional Neural Network (CNN) architecture that considers a sequence of consecutive video frames to generate a probability map of the shuttlecock’s position for each frame. This approach is aimed at learning not merely the visual features of the ball but also the distinct characteristics of its trajectory patterns in order to achieve precise positioning. The ultimate objective of this methodology is to calculate the ball’s flight path and accurately locate the shuttlecock in blurred images. The first 13 layers of TrackNet are the same backbone of the VGG16 model, and these are utilized for feature extraction. The subsequent layers, numbered 14 to 24, are inspired by DeconvNet and aim to generate predictions at the pixel level.

After obtaining the predicted coordinates of the shuttlecock, TrackNet introduces a trajectory smoothing algorithm to eliminate incorrect detections and compensate for missed detections. The entire process is divided into three steps. First, the algorithm calculates the distance between predicted coordinates in adjacent frames. If the distance exceeds 100 pixels, it is considered a prediction error and is removed. Next, a sliding window of size 7 is defined to calculate the quadratic curve of these coordinates if there are more than three predicted coordinates in this window. At this stage, any detected coordinate more than 50 pixels away from the curve is removed. Finally, if the distance between the predicted coordinate and the adjacent quadratic curves is less than 5 pixels, a final curve is obtained, which is estimated from the predicted coordinates across 15 consecutive frames. This curve is used to compensate for the missing value in these 15 frames. After this step, TrackNet checks whether there are any missing coordinates in each frame. If any missing coordinates exist, it calculates the quadratic curve through the adjacent six frames and compensates for this missing value [59]. The algorithm is described in Algorithm 1.
**Algorithm 1**: Trajectory Smoothing Algorithm**Input**: Predicted trajectory **Output**: Smoothed trajectory **Step 1: Denoising** **foreach** *coordinate* **do**
 *before_dist* ← get distance to prior frame;
 *after_dist* ← get distance to next frame;
 **if** *before_dist* ≥ 100 pixels ***or*** *after_dist* ≥ 100 pixels **then**
  remove coordinate as misprediction;
 **end**
**end**
**Step 2: Curve Fitting**
**foreach** *coordinate* **do**
 *before_window* ← get previous seven frames’ coordinates;
 *after_ window* ← get next seven frames’ coordinates;
 **if** *at least three coordinates in before_window* **then**
  fit quadratic curve;
  *front_dist* ← get minimum distance to the curve;
 **end**
 **if** *at least three coordinates in after_window* **then**
  fit quadratic curve;
  *back_dist* ← get minimum distance to the curve;
 **end**
 **if** *front_dist* ≥ 50 pixels ***or*** *back_dist* ≥ 50 pixels **then**
  remove coordinate as misprediction;
 **end**
**end**
**Step 3: Interpolation**
**foreach** *coordinate* **do**
 **if** *front_dist* ≤ 5 pixels ***or*** *back_dist* ≤ 5 pixels **then**
  fit quadratic curve;
  interpolate missing coordinates;
 **end**
**end**
**foreach** *frame with missing coordinate* **do**
 **if** *have three coordinates in before_window and after_window* **then**
  fit quadratic curve;
  interpolate missing coordinates;
 **end**
**end**

### 2.5. Separate Shots in a Rally

**Figure 4:**
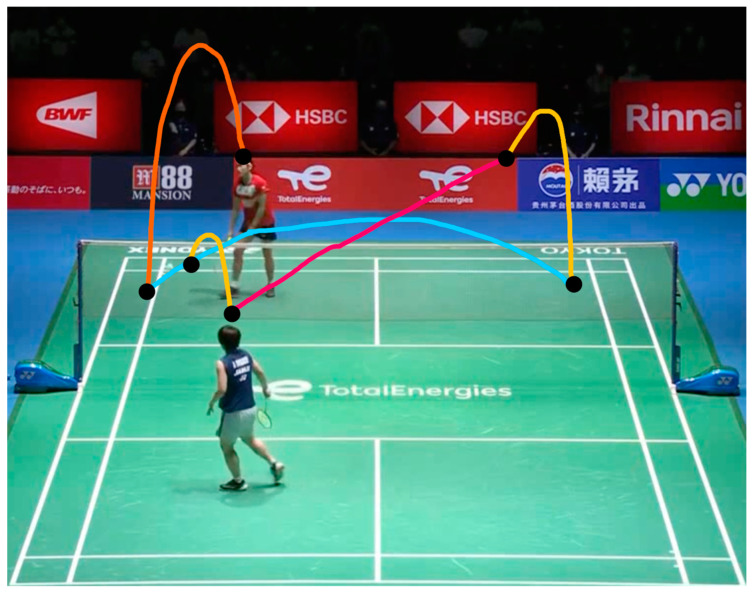
Example of the shuttlecock trajectories during a play. Different colors represent different shots. The black dots imply the hit moments.

In badminton, a rally refers to the exchange of shots while the shuttlecock is in play. Figure 4 shows the partial shuttlecock flight paths during the match between Chou Tien Chen and Anders Antonsen at the 2019 China Badminton Open, with black dots marking the shooting events. Other colors represent different shots. In observing multiple games, Huang et al. [58] found that most of the hit action occurs at the relatively low point of the trajectory of the shuttlecock, specifically at the place where the trajectory begins to rise, as shown in Figure 4. Therefore, they proposed two algorithms to identify shots, including the “Peak Identification Method” and the “Direction Identification Method”. The Peak Identification Method converts the shuttlecock position from (*x*, *y*) image coordinates to (*f_i_*, *y*), where *f_i_* represents the frame number. After this conversion, the shuttlecock’s trajectories will form a wave-like pattern and the hit moments primarily occur at the peak of this wave pattern. The frame of a hit can be identified by detecting those peaks. However, some trajectories do not exhibit obvious changes in *y*. To address this problem, TrackNet proposed the Direction Identification Method, which checks for sudden changes in the direction of the shuttlecock. If there is a significant change in the direction of the shuttlecock, it is considered a hit that forces the shuttlecock to change its flight direction.

## 3. Proposed Method

**Figure 5:**
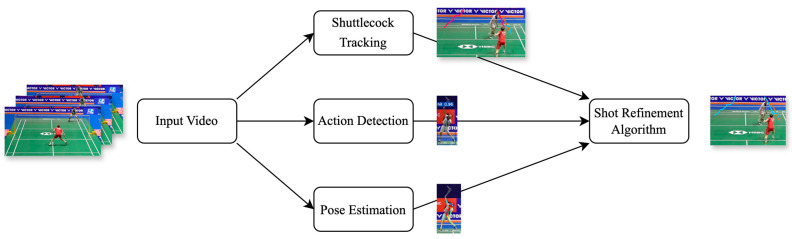
System flowchart. Different colors represent different trajectories.

The vision-based approach proposed in this study is specifically designed for single-player badminton matches. The system flowchart is illustrated in Figure 5. Initially, the Pose Estimation Module is deployed to acquire the body postures of players in each frame. Subsequently, the player’s footprints are recorded by transforming their ankle points to a plane coordinate system via perspective transformation. Meanwhile, the Badminton Tracking Module is employed to track the flight trajectory of the shuttlecock. The extracted trajectories are then processed by the Shot Refinement Module to cut out each shot. Lastly, the results derived from these modules are aggregated or classified to extract information such as shot types, players’ hitting habits, causes of point loss, and serving positions. These analytical results are then graphically represented to facilitate rapid comprehension and interpretation for both players and coaches. The following subsections provide a detailed overview of each of these modules.

### 3.1. Pose Estimation

**Figure 6:**
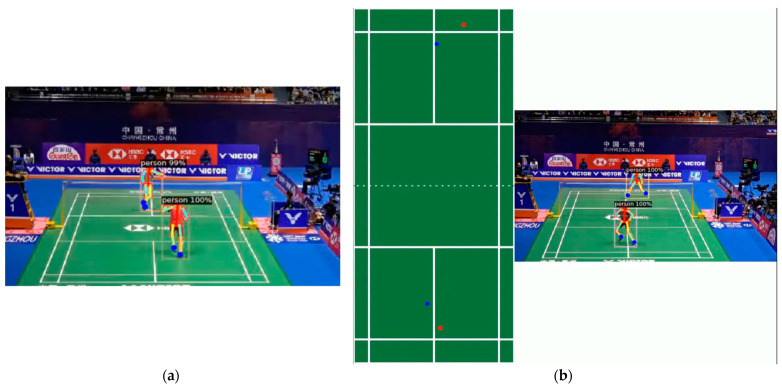
(**a**) Extracted results using Openpose; (**b**) Results of perspective transformation. Only players’ ankle points are pertained. The red dots is the left ankle points and the blue dots are the right ankle points.

Identifying the stances of badminton players is crucial for distinguishing their offensive intentions and positioning within the match. In this study, we used the DensePose model, as discussed in Section 2.2, to estimate players’ postures (see Figure 6a). Although OpenPose offers superior accuracy in human pose estimation, its computational intensity when detecting multiple individuals concurrently led us to consider DensePose as a more practical alternative.

After extracting the ankle coordinates of the players in each frame, these coordinates are projected onto an aerial view by utilizing perspective transformation (see Figure 6b). Although the presence of non-players, such as referees and spectators, can also be detected by the DensePose model, their information can be ignored because the positioning of their ankle points is outside the court. This approach ensures the accurate extraction of the players’ actual ground movement and also helps identify different players.

As mentioned in Section 2.3, since YOLO does not perform well in detecting small objects, this study only uses YOLO to detect the swinging actions of the players. In terms of detecting the trajectory of the shuttlecock, this study adopted another model that aims to detect fast-moving small objects, and this model will be detailed in the next subsection. It is worth mentioning that this study does not track the moving players, so there is no need to apply the object-tracking technique across frames. The reason for using the object-detection model is that this study believes that there should be a large number of positive detections before and after the moment a player hits the shuttlecock. Combined with the trajectory of the shuttlecock, the hit moment of the player can be more accurately determined so that we can obtain precise shots in play.

### 3.2. Shuttlecock Tracking

**Figure 7:**
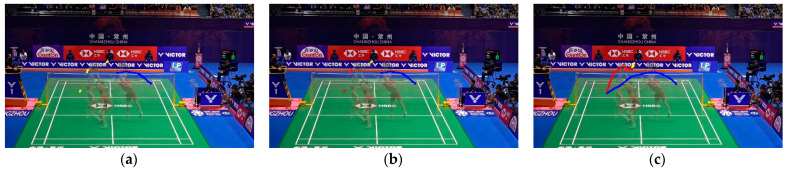
Visual comparison between TrackNet and the proposed SRA. The colored lines represent the extracted trajectories. (**a**) A wrong shot extraction due to the miss of shuttlecock detection; (**b**) The proposed SRA, which can extract correct shots under severe detection miss; (**c**) The groundtruth trajectories, which contain three shots marked in different colors.

Shuttlecocks, possessing the world record for the fastest ball type in games, with top speeds reaching 493 km/h, present significant challenges in tracking and capturing via standard optical cameras. The shuttlecock in badminton game videos is often very small and typically appears in a cluttered background, including advertising boards, court lines, and nets. Such a scenario increases the difficulty in accurately locating the shuttlecock within images. Moreover, the shuttlecock is frequently occluded by players’ bodies, adding another layer of complexity to the tracking task. To address these challenges, this study adopts the TrackNet model mentioned in Section 2.4. Since this model is specifically designed for tracking the trajectory of fast-moving balls with small volumes, it serves as the backbone of the shuttlecock tracking module in this study. However, after implementing the algorithm of TrackNet, we identified that this shuttlecock-based tracking process can be destabilized when the shuttlecock is missing. Such circumstances may result in errors in determining the actual shots between players’ swings (see Figure 7a). To address this, this study proposes a shot refinement algorithm (SRA) that integrates the detection results from the shuttlecock tracking module with the identified players in every hit to ascertain accurate shots (see Figure 7b). The details of SRA will be elaborated in the next subsection.

### 3.3. Shot Refinement Algorithm

As mentioned in the last section, when the shuttlecock is occluded by the player’s body or exits the field of view for a significant duration, it could increase the detection errors of the shuttlecock’s flight trajectory. Therefore, relying solely on the shuttlecock’s flight trajectory to determine the precise timing of a stroke might lead to substantial inaccuracies. To alleviate these unstable detections, this study proposed the shot refinement algorithm. This module incorporates an action-detection model to locate the correct timing of a player’s hit.

**Figure 8:**
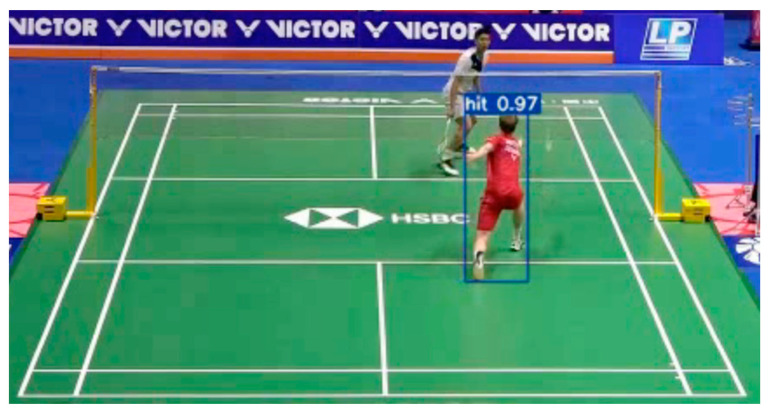
An example of hit-detection result.

To train the action-detection model, we collected a set of videos and labeled each player’s hit action manually. These videos feature multiple players performing strokes against various backgrounds, taken from official competition videos. The model used in this study was YOLOv7, as described in Section 2.3. A threshold is applied to the prediction outputs of the model, where a high confidence score in a specific frame implies that a player is performing a hit (see Figure 8). Notably, both the shuttlecock-tracking and action-detection modules are susceptible to false alarms or detection misses. The objective of the proposed SRA is to combine the results of both modules, thereby providing the precise identification of the hit moment.

Regarding the shuttlecock-tracking module, we follow the algorithm proposed by TrackNet, which includes the “Peak Identification Method” and “Direction Identification Method”, as described in Section 2.5, to detect the sudden direction change of the shuttlecock. Given this method’s reliance on the shuttlecock trajectory, the resulting detections are denoted as the Hit Detection by Trajectories ($H\mathrm{D-}T_{t}$), where *t* is the frame number. Meanwhile, the detection results derived from the YOLOv7 model yield an alternative series of players’ hit actions. As this method depends on the results of detecting the swing action, its detection results are denoted as the Hit Detection by Action (HD-A). An advantage of the action-detection module is that it can identify the player who performed the hit when considering the pose estimation results illustrated in Section 3.1. That is, each HD-A is equipped with the player’s id, denoted as $H\mathrm{D-}A_{t}^{\left(k\right)}$, where *t* is the frame number, and *k* is 1 or 2, representing the player’s id.

Initially, frames in a rally are annotated based on the HD-A results. Then, contiguous $H\mathrm{D-}A_{t}^{\left(k\right)}$ with the same *k* are merged to produce Hit Segments (*HS*). By doing so, a video sequence is partitioned into three types: $HS_{i}^{\left(1\right)}$, $HS_{i}^{\left(2\right)}$, $HS_{i}^{\left(None\right)}$, where *i* means the sequence of the segment, 1 and 2 indicate which player is executing the hit on the shuttlecock within the specified segment, and *None* means no player id is assigned for this segment.

In badminton, a shot refers to an exchange sequence between two players. That is, two consecutive detected hits should naturally be performed by players in turn. As mentioned, both *HD-T* and *HD-A* cannot avoid producing detection misses or false detections. Therefore, the intuition of SRA is to combine the outputs of both modules and produce fine-grained results. For example, given two consecutive *HD-T*s, depicted as $H\mathrm{D-}T_{t}$ and $H\mathrm{D-}T_{t^{\prime }}$, if these hits are detected within the same *HS*, meaning that they are executed by the same player, it suggests a detection error, considered to be a false positive that necessitates corrective action. Conversely, if these hits correspond to different temporal segments, it is regarded as a correct detection, subsequently considering the trajectory from $H\mathrm{D-}T_{t}$ to $H\mathrm{D-}T_{t^{\prime }}$ as a shot. Regarding all possible combinations of *HD-T* and *HS*, the SRA divides the shots into five distinct scenarios and finds the hit moments (*HMs*) as follows:

- Case 1: Only one HD-T in $HS_{i}^{\left(k\right)}$, *k* = 1 or 2

**Figure 9:**
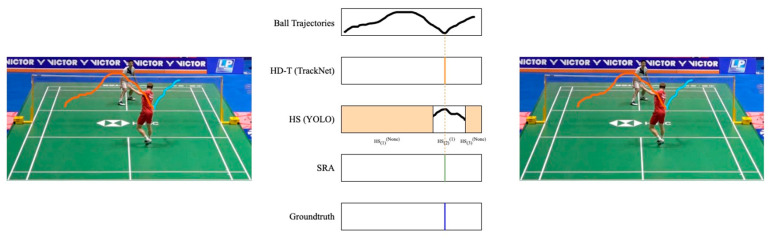
An example of case 1. The colored lines represent the extracted trajectories. There is only one *HD-T* in a hit sequence, so it is considered a true hit moment.

This scenario is considered as a correct detection (see Figure 9). Given that a single swing action can only result in one hitting time point, set $H\mathrm{D-}T_{t}$ as $HM_{t}^{\left(k\right)}$, where *k* is the same as the *k* of $HS_{i}^{\left(k\right)}$.

- Case 2: More than one HD-T in $HS_{i}^{\left(k\right)}$, *k* = *None*

**Figure 10:**
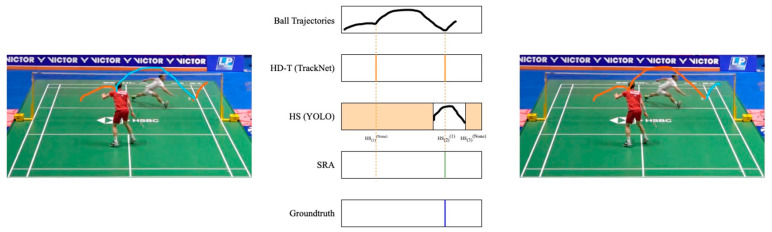
An example of case 2. The colored lines represent the extracted trajectories. *HD-T* occurs in a hit sequence that does not belong to any player, so it is considered a false positive and is removed.

This scenario is classified as a false positive (see Figure 10), as it is a time segment that should not contain any hitting action. Consequently, all *HD-T* occurrences within this segment are eliminated.

- Case 3: Multiple HD-T in $HS_{i}^{\left(k\right)}$, *k* = 1, 2

**Figure 11:**
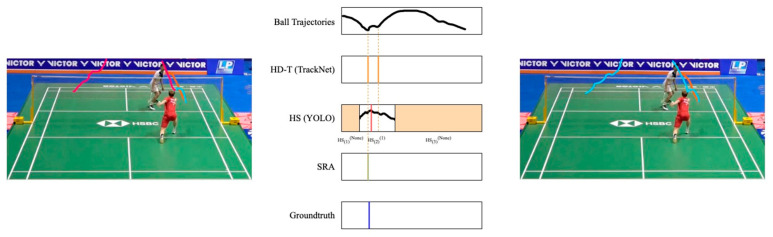
An example of case 3. The colored lines represent the extracted trajectories. Multiple *HD-T*s occurr in a hit sequence which belongs to a player, so only one *HD-T* is retained.

This scenario represents a concurrent occurrence of correct detection and false positives (see Figure 11), given that only a single hit moment can exist within a swing action. Therefore, the frame with the highest confidence score in $HS_{i}^{\left(k\right)}$ is initially identified. Subsequently, set the $H\mathrm{D-}T_{t}$ closest to this frame as $HM_{t}^{\left(k\right)}$, while the others are disgarded.

- Case 4: No HD-T in $HS_{i}^{\left(k\right)}$, *k* = 1, 2

**Figure 12:**
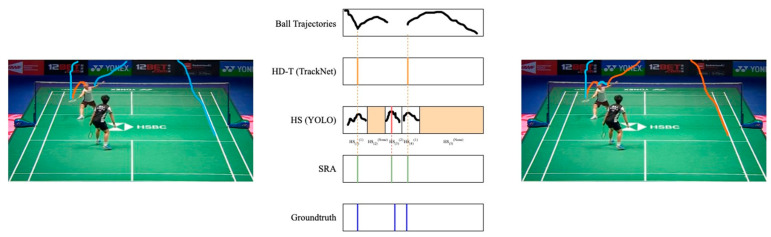
An example of case 4. The colored lines represent the extracted trajectories. No *HD-T* exists in a hit sequence that belongs to a player. In this situation, the frame that provides highest confidence by the detection model is considered the hit moment.

This instance is classified as a detection miss or false negative because a swing action should result in a hit (see Figure 12). Therefore, within this segment, the frame exhibiting the highest confidence score within $HS_{i}^{\left(k\right)}$ is selected as $HM_{t}^{\left(k\right)}\;$to account for the absent *HD-T*.

- Case 5: No HD-T in $HS_{i}^{\left(k\right)}$, *k* = *None*

**Figure 13:**
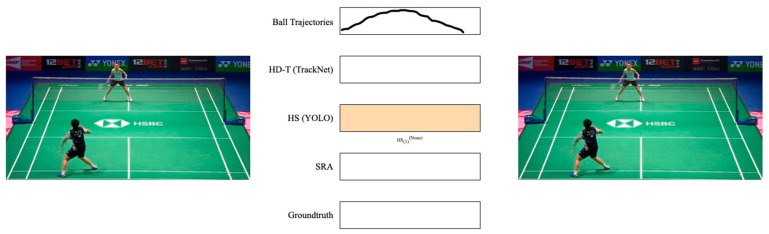
An example of case 5. No *HD-T* exists in a hit sequence that does not belong to any player. This situation is considered true negative.

This scenario is categorized as an accurate detection, denoted as a true negative (see Figure 13). During this time segment, the shuttlecock is observed to be in flight while no players are in the process of hitting, consequently resulting in the absence of *HD-T*.

We summarize the proposed SRA and illustrate its flow in Algorithm 2.
**Algorithm 2**: Shot Refinement Algorithm**Input**: *HD-T*, *HS* **Output**: True Hit Moment (THM)$∶=\left\{HM_{t}^{\left(p\right)}\right\}$
Initialize: i←0 (current HS sequence)
**while** i < total HS sequence **do**
 *p* ← *k* of $HS_{i}^{\left(k\right)}$;
 **switch** *condition* **do**
   **case** *only one HD-T in* $HS_{i}^{\left(k\right)}$, *k* = 1 *or* 2 **do**
     *t* ← *t* **of** $H\mathrm{D-}T_{t}$;
     add $HM_{t}^{\left(p\right)}$ to THM;
   **end**
   **case** *more than one HD-T in* $HS_{i}^{\left(k\right)}$, *k* = *None* **do**
     THM is not assigned;
   **end**
   **case** *multiple HD-T in* $HS_{i}^{\left(k\right)}$, *k* = 1 *or* 2 **do**
     *t* ← the frame of the player with the highest confidence in $H\mathrm{D-}T_{t}$;
     add $HM_{t}^{\left(p\right)}$ to THM;
   **end**
   **case** *no HD-T in* $HS_{i}^{\left(k\right)}$, *k* = 1 *or* 2 **do**
     *t* ← the frame with the highest confidence score in $HS_{i}^{\left(k\right)}$;
     add $HM_{t}^{\left(p\right)}$ to THM;
   **end**
   **case** *no HD-T in* $HS_{i}^{\left(k\right)}$, *k* = *None* **do**
     do nothing;
   **end**
 **end**
 *i* ← *i* + 1;
**end**

This study includes a comparison of the accuracy of different methods to verify the effectiveness of the proposed SRA. The experimental results are discussed in the next section.

## 4. Experimental Results and Discussions

In this section, several experiments are conducted to demonstrate the validity of the proposed approach. All experiments were conducted on a 10th generation Core i9 Intel CPU with 32 GB of RAM and an RTX 3080Ti GPU. The algorithm was implemented using Pyton 3.8 with tensorflow 2.12.

### 4.1. Data Acquisition

**Figure 14:**
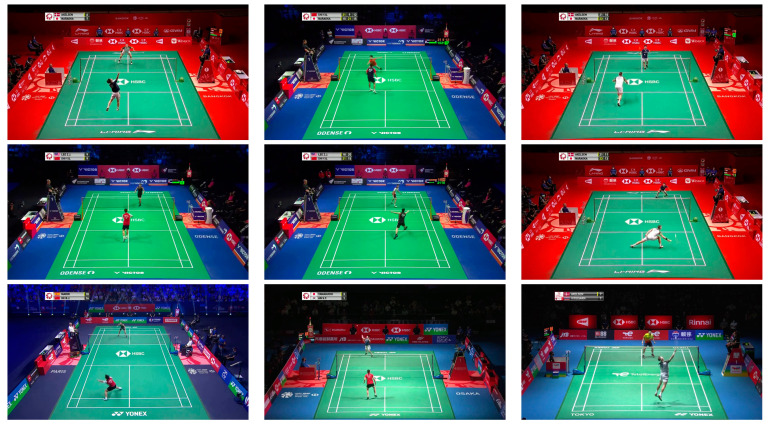
Some sample images from BWF official videos.

This study uses two datasets for experiments. The first dataset was obtained from publicly accessible videos provided by the Badminton World Federation (BWF) on YouTube, comprising 32 matches and 602 rallies across 8975 images. The second dataset was from the public dataset of the National University Competition in Artificial Intelligence, sponsored by the Ministry of Education, Taiwan. This dataset comprised 98,675 images from 354 matches and 6855 rallies. Overall, the datasets included 16 players, with a gender distribution of 7 females and 9 males. Figure 14 shows some sample images from BWF official videos.

### 4.2. Comparison with State of the Art

In this study, we evaluated the shot extraction performance using two methods: the trajectory-based detection, which is the method proposed by TrackNet, and our proposed method, SRA. For the first method, we reimplemented the algorithm and adopted the same threshold setting as the original study. We adopted the temporal Intersection over Union (t-IoU) as the metric (see Equation (1)):

(1) $$t\mathrm{-IoU}=\frac{HM_{i}^{(1↔2)}\cap GT}{HM_{i}^{(1↔2)}\cup GT},$$

where *i* is the *i*th hit moment, and *GT* is the groundtruth shots. The terminology 1↔2 means a transition from different players, indicating that this temporal segment is a shot. Note that the shots are always continuous, so each predicted shot may intersect with two adjacent groundtruth shots. In that case, we only consider the groundtruth shot with a higher IoU. The precision and recall of the model are calculated using the metric depicted in Equations (2) and (3).

(2) $$\mathrm{Precision}=\frac{\#\;\mathrm{of}\;\mathrm{true}\;\mathrm{detections}}{\mathrm{all}\;\mathrm{detections}}$$

(3) $$\mathrm{Recall}=\frac{\#\;\mathrm{of}\;\mathrm{true}\;\mathrm{detections}}{\mathrm{all}\;\mathrm{ground}\;\mathrm{truths}}$$

A detected shot is considered as a true detection if t-IoU is above a threshold. As shown in Table 1, the trajectory-based method only yielded a precision of 0.588, a recall rate of 0.936, and an F1 score of 0.723 when the IoU threshold was set to 0.5. In contrast, the proposed SRA resulted in a significant improvement in extracting accurate shots, with the precision increasing to 0.843, a slightly lower recall rate of 0.882, and the F1 score reaching 0.862. No matter the chosen of threshold, the proposed SRA exhibits better results than the TrackNet method.

The primary objective of the algorithm is to eliminate false positives generated by *HD-T* with the assistance of *HS*. We observe that the *HD-T* results align more closely with the true hit moment compared to those obtained from *HS*. Therefore, the detection results from case 2 and 3 in SRA are likely more precise than those from case 4 in SRA. In order to minimize the occurrence of case 4, we attempted to increase the number of detections of the *HD-T* model. Although this tuning decreased the overall performance of *HD-T*, the final results can be recovered by the process in case 2 and 3 in SRA. To verify this approach, we conducted a comparative analysis of two combinations: *HD-T* (TrackNet) and *HD-T* (TrackNet) + SRA, as well as *HD-T* (tuned) and *HD-T* (tuned) + SRA. As shown in Table 1, the precision of the *HD-T* (tuned) decreased to 0.524, and the recall also decreased to 0.897, but with the help of SRA, the precision bounced back to 0.897, reflecting an increase of 37.3%. Additionally, both the recall rate and the F1 score exhibited remarkable improvements. The results demonstrate that the SRA performance can be further improved with a tuned *HD-T*. Because the proposed method includes an additional YOLO model, the overall processing time is increased. Even though the processing time of SRA is longer than using TrackNet alone, we believe this trade-off is worthwhile.

**Figure 15:**
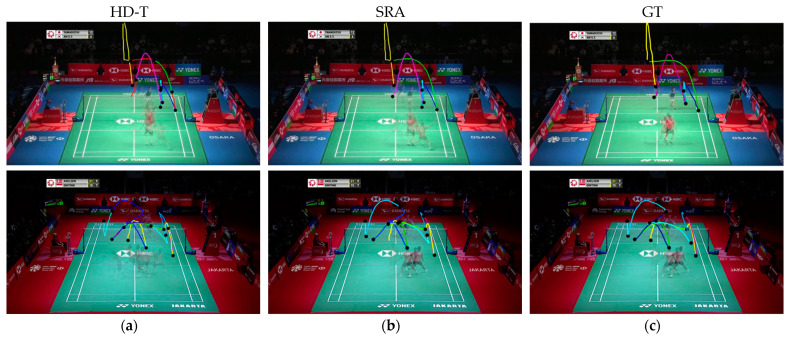
Trajectory-extraction results. Each column represents the extraction results of a method: (**a**) HD-T; (**b**) SRA; (**c**) Groundtruth. Black dots represent the hit moment and the colored lines represent the extracted trajectories. The proposed SRA can effectively address the false positives produced by HD-T.

Figure 15 allows us to clearly see that SRA can successfully eliminate false positives generated by HD-T. As shown in the first row of Figure 15, HD-T produced a false positive due to an error in detecting the shuttlecock’s trajectory (indicated by the red segment). By using SRA, we can confirm that no other player was performing a hitting action during that time, thereby successfully eliminating that false positive.

### 4.3. Shot Type Classification

An advantage of recognizing the shuttlecock’s trajectory is that it can be used to distinguish the type of shots. The analysis of shot types is closely related to classifying a player’s attack type. Therefore, this study also examined the effect of SRA on recognizing different types of shots.

In this study, shots are categorized into seven primary types: clear, drive, smash, net, drop, lift, and push. Notably, this study only considers a few features but demonstrates the superiority of the proposed method. The seven-dimensional features used in this model are as follows:

- Both players’ ankle positions (*x*_1_, *y*_1_, *x*_2_, *y*_2_) at the hit moment, which is obtained by the method proposed in Section 3.1.
- Flight duration of the shuttlecock, denoted as *T* in regard to the number of frames.
- The displacement of the shuttlecock (Δ*x*, Δ*y*), quantified as the difference in its positions from the start to the end of the shot.

**Figure 16:**
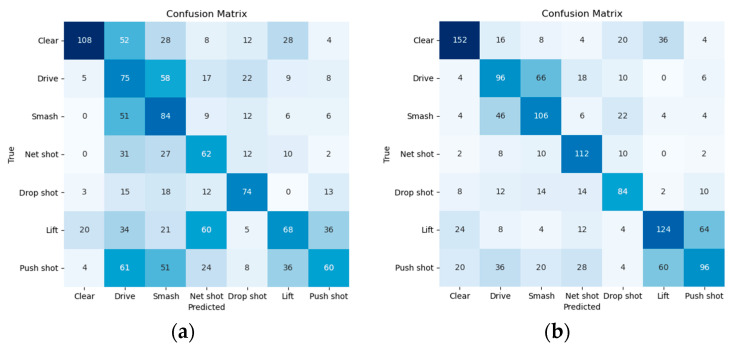
Comparison of shot-type classification results when IoU threshold is set to 0.5. A darker color represents a higher value. (**a**) TrackNet; (**b**) SRA.

This study does not consider the shuttlecock positions in every frame due to the occurrence of detection misses. However, as a complete shot can still be obtained, the shuttlecock displacement can be considered reliable. These features provide an enhanced understanding of the dynamics of the game, thereby enabling accurate classification of shot types and differentiation of playing styles. Limited by the number of features and samples available for training, this study employs the XGBoost model for shot classification. The results are presented in Table 2. In Table 2, we can see that the proposed SRA method achieves 72.1% accuracy when the IoU threshold is set to 0.95. When the threshold is loosened to 0.5, the classification accuracy drops to 54.1% but still outperforms TrackNet. The degradation happens because all features heavily rely on accurate hit moments. A looser threshold implies less precise shots. However, these results still demonstrate that SRA has a better ability to extract accurate shots compared to TrackNet. As shown in Figure 16, it is notable to acknowledge that some shot types, such as drives and smashes, exhibit similar feature values, so the classification accuracy is not as good as that of other types of shots.

## 5. Conclusions

The advancement of an automated sports data analysis system signifies a considerable progression in improving players’ skills and the refinement of coaching methodologies. In this study, videos captured from a monocular camera are employed for analyzing badminton games, thereby alleviating the necessity for players to wear any additional equipment for data collection.

Analyzing badminton shot types is beneficial in planning player’s strategies. The correctness of shot classification relies on detecting the precise shot trajectories. Previous studies have proposed shuttlecock-tracking technology to extract the trajectory of the shuttlecock and identify each shot based on trajectory alterations. Building upon their efforts, this study proposes an enhancement approach by combining the results of both shuttlecock trajectories, action detection, and pose estimation for refined hit detection. The proposed algorithm leverages the results of TrackNet, with a YOLO-based hit detection model, to mitigate the occurrence of false positives and false negatives generated during the detection process. The experimental results demonstrate that the proposed method can more accurately determine each shot, and the results benefit subsequent advanced analyses, such as shot type classification. These precise results can be further utilized to analyze more abstract concepts, such as a player’s hitting style or tactical flaws in gameplay.

Nevertheless, because only a monocular camera is utilized as the input, the proposed system is constrained by a single perspective, making it difficult to correctly project 2D image coordinates back to 3D real coordinates. This may potentially affect the accuracy of ankle coordinates and lead to misjudgments of the player’s position, thereby impacting the analysis of shot types and player movement direction. For example, when the player jumps and performs a power strike, the results of the inferred ankle position are incorrect. However, this scenario does not occur very often, hence, the approach still achieves satisfactory results in terms of the shot-type classification. We acknowledge that applying deep learning-based models could potentially improve shot-type classification performance. However, these models are often seen as data-hungry, and the data collected in this study may not be sufficient to effectively train such models. Future researchers can focus on obtaining accurate shuttlecock and players’ 3D real-world ankle coordinates. By doing so, it is expected that the accuracy of trajectory extraction can be further improved.

## Figures and Tables

**Table 1:** Comparison between the proposed SRA and TrackNet. Numbers in bold represent the highest value (higher is better).

Method	Processing Time (Seconds)	IoU Threshold	Precision	Recall	F1
*HD-T* (TrackNet)	0.032	0.5	0.588	**0.936**	0.723
		0.85	0.131	0.209	0.162
		0.95	0.046	0.073	0.056
*HD-T* (TrackNet) + SRA	0.174	0.5	0.843	0.882	0.862
		0.85	0.435	0.455	0.444
		0.95	0.244	0.255	0.249
*HD-T* (tuned)	0.032	0.5	0.524	0.897	0.661
		0.85	0.084	0.153	0.101
		0.95	0.038	0.069	0.049
*HD-T* (tuned) + SRA	0.175	0.5	**0.897**	0.913	**0.905**
		0.85	0.461	0.483	0.472
		0.95	0.269	0.271	0.270

**Table 2:** Shot-type classification accuracy with different IoU thresholds.

Method	IoU Threshold	Accuracy
TrackNet	0.5	0.388
	0.85	0.610
	0.95	0.719
SRA	0.5	0.541
	0.85	0.654
	0.95	0.721

## Data Availability

Restrictions apply to the availability of these data. Data were obtained from https://aidea-web.tw (accessed on 29 May 2024) and are available at https://aidea-web.tw/topic/cbea66cc-a993-4be8-933d-1aa9779001f8?lang=en (accessed on 29 May 2024) with the permission of the authors of [59].

## References

[1] Zhang J., Piao Z.H., Bai D.H. (2022). Application Analysis of Badminton Intelligence based on Knowledge Graphs. Tob. Regul. Sci. (TRS).

[2] Lin W.B., Yang S.C., Chen M.Y. (2021). Know thyself, know thy enemy, a thousand battles, a thousand victories—The data science in baseball intelligence gathering for technical and tactical analysis. Q. Chin. Phys. Educ..

[3] Lin W.B., Yeh S.W., Yang C.W. (2017). A Study of efficiency management for players and teams in CPBL from the viewpoint of data science. Phys. Educ. J..

[4] Cullen F.T., Myer A.J., Latessa E.J. (2009). Eight lessons from moneyball: The high cost of ignoring evidence-based corrections. Vict. Offenders.

[5] Trackman. https://www.trackman.com/baseball.

[6] Marsten H. What is TrackMan Data?. https://trackman.zendesk.com/hc/en-us/articles/115002776787-What-is-TrackMan-Data.

[7] Rapsodo Design the Perfect Pitch. Develop a Complete Pitch Arsenal with Industry Leading Data..

[8] Rapsodo Rapsodo & Driveline Create Official Analytics Partnership. https://finance.yahoo.com/news/rapsodo-driveline-baseball-announce-partnership-160146669.html.

[9] Huang J., Hsu H.J. (2020). Approximating strike zone size and shape for baseball umpires under different conditions. Int. J. Perform. Anal. Sport.

[10] Yığman C. (2018). Predictive Analysis of Successful Basketball Shots: The Euroleague Case. Unpublished Doctoral Dissertation.

[11] Miller T. (2015). Sports Analytics and Data Science: Winning the Game with Methods and Models.

[12] Sarlis V., Tjortjis C. (2020). Sports analytics—Evaluation of basketball players and team performance. Inf. Syst..

[13] Sharef N.M., Mustapha A., Nor Azmi M.B., Nordin R. Basketball Players Performance Analytic as Experiential Learning Approach in Teaching Undergraduate Data Science Course. Proceedings of the International Conference on Advancement in Data Science, E-learning and Information Systems (ICADEIS).

[14] Chang Y.C., Lin Y.H. (2020). A study of basketball analytics. Q. Chin. Phys. Educ. Soc. Phys. Educ..

[15] SportVU Tracking System Could Find Reach beyond Court For NBA, Teams. https://www.sportsbusinessjournal.com/Daily/Issues/2013/10/29/NBA-Season-Preview/Tech.aspx.

[16] Wang K.C., Zemel R. Classifying NBA offensive plays using neural networks. Proceedings of the MIT Sloan sports analytics Conference.

[17] Rajiv S., Romijnders R. (2016). Applying deep learning to basketball trajectories. arXiv.

[18] Cao C. (2012). Sports Data Mining Technology Used in Basketball Outcome Prediction. Master’s Thesis.

[19] Cohan A., Schuster J., Fernandez J. (2021). A Deep Learning Approach to Injury Forecasting in NBA Basketball. J. Sports Anal..

[20] Valter D.S., Adam C., Barry M., Marco C. (2006). Validation of Prozone®: A new video-based performance analysis system. Int. J. Perform. Anal. Sport.

[21] Liu H., Hopkins W., Gómez A.M., Molinuevo S.-J. (2013). Inter-operator reliability of live football match statistics from OPTA Sportsdata. Int. J. Perform. Anal. Sport.

[22] Deltatre. https://www.deltatre.com.

[23] Reep C., Benjamin B. (1968). Skill and Chance in Association Football. J. R. Stat. Soc. Ser. A (Gen.).

[24] Reep C., Pollard R., Benjamin B. (1971). Skill and Chance in Ball Games. J. R. Stat. Soc. Ser. A (Gen.).

[25] Takahashi M., Yamanouchi Y., Nakamura T. Real-Time Ball Position Measurement for Football Games Based on Ball’s Appearance and Motion Features. Proceedings of the 11th International Conference on Signal-Image Technology & Internet-Based Systems (SITIS).

[26] Stoeve M., Schuldhaus D., Gamp A., Zwick C., Eskofier B.M. (2021). From the Laboratory to the Field: IMU-Based Shot and Pass Detection in Football Training and Game Scenarios Using Deep Learning. Sensors.

[27] Rahman M.A. (2020). A deep learning framework for football match prediction. SN Appl. Sci..

[28] Merhej C., Beal R.J., Matthews T., Ramchurn S. What Happened Next? Using Deep Learning to Value Defensive Actions in Football Event-Data. Proceedings of the 27th ACM SIGKDD Conference on Knowledge Discovery & Data Mining.

[29] Chen C.H. (2022). Analysis of the technical aspects of the men’s singles badminton final eight in 2020 Tokyo Olympics. J. Badminton Sports Taiwan.

[30] Josue F., Abdullah M.F., Zulkapri I., Soeed K., Tariq I. (2020). Movement pattern in term of court coverage among top international male and female badminton players during BWF World Championships 2013. J. Sains Sukan Pendidik. Jasm..

[31] Sharma M., Kumar M.N., Kumar P. (2021). Badminton match outcome prediction model using Naïve Bayes and Feature Weighting technique. J. Ambient. Intell. Humaniz. Comput..

[32] Cheng Y.P., Tsai T.H., Tsai T.C., Chiu Y.H., Chu H.K., Hu M.C. OmniScorer: Real-Time Shot Spot Analysis for Court View Basketball Videos. Proceedings of the ACM Multimedia Asia 2023.

[33] Liu P.X., Pan T.Y., Hu M.C., Chu H.K., Lin H.S., Hsieh W.W., Cheng C.-J. An Exploratory Investigation into the Design of a Basketball Immersive Vision Training System. Proceedings of the 2023 IEEE Conference on Virtual Reality and 3D User Interfaces Abstracts and Workshops (VRW).

[34] Wang W.Y., Chang K.S., Chen T.F., Wang C.C., Peng W.C., Yi C.W. (2020). Badminton Coach AI: A badminton match data analysis platform based on deep learning. Phys. Educ. J..

[35] Martínez G.H. (2019). Openpose: Whole-Body Pose Estimation. Ph.D. Dissertation.

[36] Güler R.A., Neverova N., Kokkinos I. Densepose: Dense human pose estimation in the wild. Proceedings of the IEEE Conference on Computer Vision and Pattern Recognition.

[37] Wu Y., Kirillov A., Massa F., Lo W.Y., Girshick R. Detectron2: A PyTorch-Based Modular Object Detection Library. https://ai.facebook.com/blog/-detectron2-a-pytorch-based-modular-object-detection-library-/.

[38] Vuola A.O., Akram S.U., Kannala J. Mask-RCNN and U-net ensembled for nuclei segmentation. Proceedings of the 2019 IEEE 16th International Symposium on Biomedical Imaging (ISBI 2019).

[39] Güler R.A., Trigeorgis G., Antonakos E., Snape P., Zafeiriou S., Kokkinos I. Densereg: Fully convolutional dense shape regression in-the-wild. Proceedings of the IEEE Conference on Computer Vision and Pattern Recognition.

[40] Luo W., Xing J., Milan A., Zhang X., Liu W., Kim T.K. (2021). Multiple object tracking: A literature review. Artif. Intell..

[41] Bewley A., Ge Z., Ott L., Ramos F., Upcroft B. Simple online and realtime tracking. Proceedings of the 2016 IEEE International Conference on Image Processing (ICIP).

[42] Wojke N., Bewley A., Paulus D. Simple online and realtime tracking with a deep association metric. Proceedings of the 2017 IEEE International Conference on Image Processing (ICIP).

[43] Lin S.D., Chang T., Chen W. (2021). Multiple Object Tracking using YOLO-based Detector. J. Imaging Sci. Technol..

[44] Krishna N.M., Reddy R.Y., Reddy M.S., Madhav K.P., Sudham G. Object Detection and Tracking Using Yolo. Proceedings of the 2021 Third International Conference on Inventive Research in Computing Applications (ICIRCA).

[45] Megalingam R.K., Babu D.H.T.A., Sriram G., YashwanthAvvari V.S. Concurrent detection and identification of multiple objects using YOLO algorithm. Proceedings of the 2021 XXIII symposium on image, signal processing and artificial vision (STSIVA).

[46] Redmon J., Divvala S., Girshick R., Farhadi A. You only look once: Unified, real-time object detection. Proceedings of the IEEE Conference on Computer Vision and Pattern Recognition (CVPR).

[47] Redmon J., Farhadi A. YOLO9000: Better, faster, stronger. Proceedings of the IEEE Conference on Computer Vision and Pattern Recognition (CVPR).

[48] Redmon J., Farhadi A. (2018). Yolov3: An incremental improvement. arXiv.

[49] Bochkovskiy A., Wang C.Y., Liao H.Y.M. (2020). Liao. Yolov4: Optimal speed and accuracy of object detection. arXiv.

[50] Li C., Li L., Jiang H., Weng K., Geng Y., Li L., Ke Z., Li Q., Cheng M., Nie W. (2022). YOLOv6: A single-stage object detection framework for industrial applications. arXiv.

[51] Wang C.Y., Yeh I.H., Liao H.Y.M. (2024). You Only Learn One Representation: Unified Network for Multiple Tasks. J. Inf. Sci. Eng..

[52] Wang C.Y., Bochkovskiy A., Liao H.Y.M. YOLOv7: Trainable Bag-of-Freebies Sets New State-of-the-Art for Real-Time Object Detectors. Proceedings of the 2023 IEEE/CVF Conference on Computer Vision and Pattern Recognition (CVPR).

[53] Tan L., Dong X., Ma Y., Yu C. A Multiple Object Tracking Algorithm Based on YOLO Detection. Proceedings of the 2018 11th International Congress on Image and Signal Processing, BioMedical Engineering and Informatics (CISP-BMEI).

[54] Cao Z., Liao T., Song W., Chen Z., Li C. (2021). Detecting the shuttlecock for a badminton robot: A YOLO based approach. Expert Syst. Appl..

[55] Li W., Liu X., An K., Qin C., Cheng Y. (2023). Table Tennis Track Detection Based on Temporal Feature Multiplexing Network. Sensors.

[56] Zhang Z., Wu F., Qiu Y., Liang J., Li S. Tracking Small and Fast Moving Objects: A Benchmark. Proceedings of the Asian Conference on Computer Vision.

[57] Wu C.W., Shieh M.D., Lien J., Yang J.F., Chu W.T., Huang T.H., Hsieh H.C., Chiu H.T., Tu K.C., Chen Y.T. (2022). Enhancing Fan Engagement in a 5G Stadium with AI-Based Technologies and Live Streaming. IEEE Syst. J..

[58] Huang Y.C., Liao I.N., Chen C.H., İk T.U., Peng W.C. TrackNet: A deep learning network for tracking high-speed and tiny objects in sports applications. Proceedings of the 2019 16th IEEE International Conference on Advanced Video and Signal Based Surveillance (AVSS).

[59] Lin Y.C. (2021). Badminton Trajectory Detection and Analysis. Master’s Thesis.

